# The combination of transcriptomics and informatics identifies pathways targeted by miR-204 during neurogenesis and axon guidance

**DOI:** 10.1093/nar/gku498

**Published:** 2014-06-04

**Authors:** Ivan Conte, Stefania Merella, Jose Manuel Garcia-Manteiga, Chiara Migliore, Dejan Lazarevic, Sabrina Carrella, Raquel Marco-Ferreres, Raffaella Avellino, Nathan Paul Davidson, Warren Emmett, Remo Sanges, Nicholas Bockett, David Van Heel, Germana Meroni, Paola Bovolenta, Elia Stupka, Sandro Banfi

**Affiliations:** 1 Telethon Institute of Genetics and Medicine, Via Pietro Castellino, 111, 80131 Naples, Italy; 2 Center For Translational Genomics and Bioinformatics, San Raffaele Scientific Institute, Via Olgettina, 58, 20132 Milan, Italy; 3 CBM Scrl, c/o Area Science Park, Basovizza, 30143 Trieste, Italy; 4 Centro de Biología Molecular ‘Severo Ochoa’, CSIC-UAM, c/Nicolas Cabrera 1, Madrid 28049, Spain; 5 CIBER de Enfermedades Raras (CIBERER), c/ Nicolas Cabrera 1, Madrid 28049, Spain; 6 UCL Cancer Institute, Huntley Street, University College London, London WC1E 6BT, UK; 7 Stazione Zoologica Anton Dohrn, Villa Comunale, 80121 Napoli, Italy; 8 Blizard Institute, Barts and The London School of Medicine and Dentistry, Queen Mary University of London, London E1 2AT, UK; 9 Medical Genetics, Department of Biochemistry, Biophysics and General Pathology, Second University of Naples, 80138 Naples, Italy

## Abstract

Vertebrate organogenesis is critically sensitive to gene dosage and even subtle variations in the expression levels of key genes may result in a variety of tissue anomalies. MicroRNAs (miRNAs) are fundamental regulators of gene expression and their role in vertebrate tissue patterning is just beginning to be elucidated. To gain further insight into this issue, we analysed the transcriptomic consequences of manipulating the expression of miR-204 in the Medaka fish model system. We used RNA-Seq and an innovative bioinformatics approach, which combines conventional differential expression analysis with the behavior expected by miR-204 targets after its overexpression and knockdown. With this approach combined with a correlative analysis of the putative targets, we identified a wider set of miR-204 target genes belonging to different pathways. Together, these approaches confirmed that miR-204 has a key role in eye development and further highlighted its putative function in neural differentiation processes, including axon guidance as supported by *in vivo* functional studies. Together, our results demonstrate the advantage of integrating next-generation sequencing and bioinformatics approaches to investigate miRNA biology and provide new important information on the role of miRNAs in the control of axon guidance and more broadly in nervous system development.

## INTRODUCTION

MicroRNAs (miRNAs), a class of short non-coding RNAs (∼22 nucleotides in length), have emerged as important regulators of gene expression in development (1). At the molecular level, they exert their function in animal cells by binding, with imperfect base pairing, to target sites in the 3′ un-translated regions (3′UTRs) of messenger RNAs. This binding either causes the inhibition of translational initiation or leads to messenger RNA (mRNA) degradation (2–5). Each miRNA is estimated to regulate, on average, the expression of 100–200 distinct target genes, so that the whole miRNA apparatus seems to participate in the control of gene expression for a significant proportion of the vertebrate transcriptome (6,7). Therefore, miRNAs are as important as transcription factors or signaling molecules in controlling cellular processes. Recent discoveries have, indeed, revealed a model in which miRNA regulatory events are woven into the known transcription factor and signaling networks that control cell fate and differentiation, modulating their activity through positive and negative feedback loops to promote programs that define the fate and character of developing cells (8–14). In humans, deregulation of miRNA expression caused by mutations in either the miRNA itself or in its target gene(s) has been correlated with a number of pathological conditions such as diabetes, neurodegenerative diseases, heart failure, hereditary deafness, among others (15–19), but the mechanistic role played by miRNAs in the underlying biological network is often unclear. Typically, miRNA function is predicted by assessing whether the predicted targets of a given miRNA are enriched for particular functional annotations. However, the limited number of *bona fide* miRNA targets that have been experimentally validated so far hamper this approach. Recently, we and others have reported that miRNAs may control functional pathways in cells by targeting, in a coordinated manner, sets of functionally correlated genes thus making co-expression analysis a valid tool to gain insight into miRNA function (20,21).

Advances in high-throughput sequencing technologies are having an immense impact on genomics (22), transcriptomics (23) and proteomics (24). Speed and accuracy of data generated have made next-generation sequencing a powerful tool to study biological events at the nucleic acid level. Here we apply mRNA deep sequencing (RNA-Seq) to gain comprehensive understanding of transcriptional processes occurring during alterations of miRNA activity. We focused our work on miR-204 for which we recently obtained functional information in Medaka fish (*Oryzias latipes*) (9,25,26). MiR-204 is highly expressed in the eye where it modulates the expression of the *Meis2* and *Ankrd13a* genes with a consequent significant impact on eye morphogenesis and differentiation (9,25). In the present study, we describe the mRNA expression profiling in the context of the alteration of miR-204 activity in Medaka fish using whole embryos. We deployed an innovative approach to the analysis of the RNA-Seq data in both miR-204 knock-down (KD) and over-expression (OE) Medaka models. This approach takes into account the different expression behavior expected of targets in KD and OE in a whole embryo context. Integrating this approach with a bioinformatics prediction of targets and with co-expression correlation analysis with respect to putative targets, we were able to identify known and novel miR-204 target gene pathways. In particular, we demonstrated *in vivo* that altered expression of miR-204 is associated with abnormal axonal projection of retinal ganglion cells to the brain. The data generated, moreover, provide the first reported RNA-Seq-based transcriptome landscape of Medaka fish, a vertebrate model of increasing interest in developmental biology (27,28), thus building a valuable resource for Medaka developmental biology, functional genomics and genome re-annotation.

## MATERIALS AND METHODS

### Medaka stocks, miR-204 duplex and morpholino injections

The Cab-strain of wild-type (WT) Medaka fish (*O. latipes*) and *Ath5*:GFP Medaka transgenic line (29) were kept as previously described (30). Embryos were staged according to (31). Morpholinos (Gene Tools, LLC, OR, USA) and miRIDIAN Dharmacon microRNA mimics were injected into one-cell fertilized embryos as described (9). Morpholinos were designed against the 5′UTR of *olEphB2* (Mo-EphB2; 5′- GTC ACT TAA GGA GCC CAG ACA TTC A – 3′) and the donor splice site of *olEfnb3* exon2 (Mo-Efnb3; 5′ GAG GCT CAC CGA TGA TGT AGT AGT C-3′), respectively. The specificity and inhibitory efficiencies of both morpholinos were determined as previously described (9). Mo-EphB2 and Mo-Efnb3 were co-injected with Mo-miR-204 at 0.015 mM and 0.06 mM concentrations into one blastomere at the one/two-cell stage, respectively. *In vitro* synthesis of the *EphB2a* full-length coding mRNAs was performed as described (9). *EphB2* mRNAs were injected at 10–50 ng/μl. The selected working concentration was 50 ng/μl. Control embryos were injected with 50 ng/μl of eGFP mRNA.

### RNA extraction, library preparation and sequencing

Total RNA from Stage 24 [St.24; 1 day and 20-h post-fertilization (pf) corresponding to E.12.5dpc in mouse] WT and injected embryos was extracted using TRIZOL (Invitrogen) according to the manufacturer's instructions and used for subsequent RNA-Seq-based profiling (32). The RNA samples were treated with 2U DNase I (Qiagen) per microgram RNA sample at 37°C for 10 min. The digested samples were then treated with 20 mg/ml proteinase K (Sigma Aldrich) at 37°C for 45 min. The quality and quantity of total RNA were assessed with the bioanalyzer 2100 (Agilent) and by optical density (Nanodrop ND-1000 spectrophotometer). Only the samples with RNA integrity number (RIN) above 8 were considered worthy for the further steps. For the spectrophotometric analysis, instead, a ribosomal RNA 28s/18s ratio between 1.8 and 2.0 was considered suitable for the subsequent phases. The RNA-Seq library was generated following the standard Illumina RNA-Seq protocol and sequenced using an Illumina HiSeq 2000 at the Center for Translational Genomics and Bioinformatics at San Raffaele Scientific Institute.

### cDNA preparation and real-time PCR

At least 250 embryos were pooled in each assay. Complementary DNA (cDNA) synthesis was performed using the SuperScript III First-Strand Synthesis System for RT-PCR using random hexamers (Invitrogen). Quantitative reverse transcriptase-polymerase chain reaction (qRT-PCR) was performed as previously described (9). Each assay was performed in triplicate. Primers used for qRT-PCR are listed in Supplementary Table S1.

### Analysis of RNA-Seq datasets

The RNA-Seq paired-end sequence reads were mapped to the Medaka genome (UCSC oryLat2) using SOAPSplice with default parameters. The SOAPSplice (33) results were converted to transcript read counts using multiBamCov of BEDTools (34). The Ensembl annotated genome assembly (release 70) was used as a reference. This approach does not yield potential novel genes, which was beyond the scope of this study, but it allowed reliable quantification of a large portion of annotated transcripts. Differential expression analysis was performed using the Linear Model for MicroArray data (LIMMA) Bioconductor package with RNA-Seq-specific 'voom' function, and using Pearson's correlation test together with rank product statistic (35). For Linear Model for MicroArray data (LIMMA) analysis, we followed the suggestions reported in the manual for experiment with different condition and multiple replicas. For correlation analysis, we used *rp* function from RankProd Bioconductor package. The matrix of 156 putative targets and the matrix with all expressed transcripts were constructed using the normalized reads count from LIMMA 'voom' function. After the result of *rp* function, we selected those transcripts with a positive correlation, an average FC greater than 0 and a percentage of false prediction (*pfp)* value less than 0.01.

### Bioinformatics prediction of miR-204 targets

Several computational algorithms designed to predict target genes of miRNA sequences have been used. The basis for these programs is the degree of sequence complementarity between a miRNA and a target 3′UTR, including the presence of a consecutive string of base pairs at the 5′ end of the miRNA known as a 'seed' or 'nucleus'. Due to sensitivity and specificity in target prediction, we combined three bioinformatics algorithms to predict miRNA target sites: miRanda (http://www.microrna.org), TargetScan (http://www.targetscan.org/) and PicTar (http://pictar.mdc-berlin.de/). All three of these computational programs allow the researcher to enter a specific miRNA symbol and the algorithm will determine all putative mRNA targets. This approach yielded 3720 putative Medaka targets which were obtained by taking human gene targets predicted by any of these programs (i.e. the union of all the results) and searching for their orthologs in Medaka using Biomart database. Since Medaka annotation provides poor data on 3′UTRs, we performed our own 3′UTR reconstruction from the RNA-Seq data generated, allowing us to verify the conservation of the miR-204 seed sequences in the newly annotated UTRs (see Supplementary Figure S1).

### Functional enrichment analyses

Enrichment analyses were performed with the web tool DAVID at http://david.abcc.ncifcrf.gov/home.jsp using default parameters (36). The Kyoto Encyclopedia of Genes and Genomes (KEGG) is one of the most important bioinformatics databases of metabolic pathways of cells. In order to visualize enriched Kyoto Encyclopedia of Genes and Genomes (KEGG) pathways, we used the EnrichR web tool (http://amp.pharm.mssm.edu/Enrichr/).

### Plasmid constructs and Luciferase assay

Plasmids containing either the WT 3′UTR sequence or its mutated version, containing three point mutations in the seed of the predicted miR-204 target site of the human *EFNB3*, *DPYSL3*, *KIF5* and *DDX3Y* genes, and psiUx plasmid constructs containing the hsa-pre-miR-204 sequence were used in Luciferase assays, as described previously (25). Each assay was performed in triplicate, and all the results are shown as means ± SD of at least three independent assays. The primer sequences used to amplify each transcript, both WT and with mutagenized miR-204 target sites are shown in Supplementary Table S1.

### *In situ* hybridization and fluorescence imaging

Whole-mount RNA *in situ* hybridizations were performed, photographed and sectioned as described (30). Digoxigenin-labeled anti-sense and sense riboprobes for *olEfnb3* and o*lEphB2* were used. Probes were generated from cDNA amplified from st24–28 embryos using specific primers (Supplementary Table S1) and cloned into the Topo-TA-vector (Invitrogen). Ath5:GFP Medaka transgenic lines were used for cryosections, confocal image stacks were acquired to detect native GFP fluorescence using Zeiss (LSM 710).

## RESULTS

### Generation of miR-204 knock-down and over-expression models in Medaka fish

We previously demonstrated that miR-204 is required for lens differentiation and dorso-ventral patterning of the vertebrate optic cup through the modulation of several target genes (9,25,26). However, KD of miR-204 caused other eye developmental defects, including microphthalmia and alterations of lens polarity that could not be easily explained by the misregulation of the above targets. Thus, to gain further insight into the composition of miR-204-regulated gene networks and to identify yet unexplored roles of this miRNA in eye development and function, we either overexpressed miR-204 or negatively interfered with its expression using a multiblocking morpholino-based knock-down approach (37). Injection of Mo-miR-204 did not appear to affect early embryonic development before Stage 24 [St.24; 1 day and 20-h post-fertilization (pf) corresponding to E.12.5dpc in mouse], when miR-204 morphant KD showed reduced optic cup and abnormal lens morphology, as previously described (9). Notably, miR-204 OE also caused severe microphthalmia, with small lenses at St.24 and onward (9). To identify the global effects derived from the perturbations of miR-204 activity, we decided to analyse the transcriptome profiles on total RNA from WT, miR-204 KD and miR-204 OE Medaka embryos at St.24, i.e. when phenotypic alterations start to be observed with respect to control animals.

### Mapping and differential expression analysis of the Medaka transcriptome

**Figure 1.**
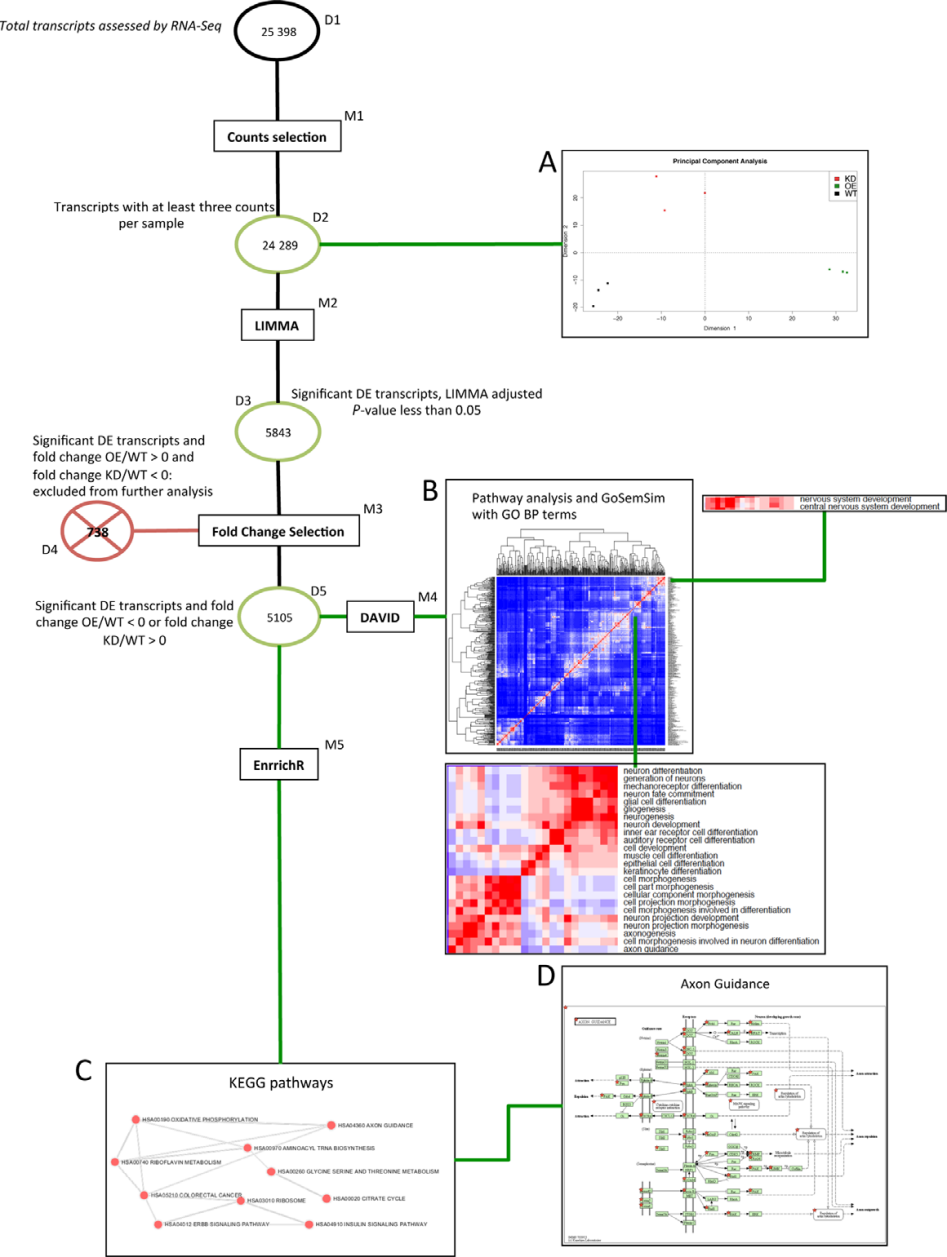
Flow chart of Differentially Gene Expression (DGE) analysis using LIMMA. Circles represent datasets used in the analysis workflow, rectangles indicate methods applied to the datasets and panels present results from specific datasets. We started our analysis from the Ensembl Medaka transcriptome of 25 398 transcripts (D1). We then applied the multiBamCov tool from BedTools to eliminate transcripts with less than nine reads across the nine replicas (M1) which yielded dataset D2. The PCA of D2 is presented in panel A. We performed differential gene expression analysis using LIMMA and 'voom' selecting transcripts with an adjusted *P*-value of less than 0.05 (M2) yielding D3. We further selected transcripts based on fold change expected of an miR-204 target, i.e. of less than 0 in the contrast OE/WT or a fold change grater than 0 for the contrast KD/WT yielding D5, and thus excluding transcripts in D4. We converted selected transcripts in human orthologous genes using Ensembl data and then performed Gene Ontology enrichment analysis on those genes using the DAVID web server (M4) using human orthologs of D2. The results from the DAVID analysis are presented using the GOSemSim clustering tool in panel B. Specific clusters in this analysis are also presented as magnified examples. Functional enrichment for KEGG pathways was also performed using the EnrichR web server (M5). The results of this analysis are presented in panel and the Axon Guidance KEGG pathway is presented in panel D, indicating differentially expressed genes within D5 with a red star.

We generated and analysed a profile of the Medaka early embryonic transcriptome using RNA-Seq. RNA-Seq libraries were prepared for each RNA sample in biological triplicates, i.e. three WT, three miR-204 KD and three miR-204 OE whole (St24) embryo RNAs. Each library was sequenced using 100 bp paired-end sequencing on the Illumina HiSeq 2000 system. The sequences were aligned to the Medaka genome using SoapSplice, a splice-aware mapping tool (33). The mappings were then converted to transcript specific read count values using multiBamCov from BedTools utilities (34). We used annotated transcripts from the Ensembl database as gene annotation for the experiment. This approach yielded read count values for a total of 25 398 annotated Medaka transcripts belonging to 20 424 Medaka genes (D1 in Figure 1). From these we excluded all transcripts that had less than nine total read counts (i.e. on average at least one read count per biological replica), yielding a total of 24 289 transcripts (D2 in Figure 1). A principal component analysis (PCA) performed on these expression values clearly differentiated the transcriptome profiles of WT animals from both miR-204 OE and KD (panel A in Figure 1 and Supplementary Figure S2).

These transcripts were analysed for differential expression between WT biological replicates and both KD and OE using the LIMMA package and the RNA-Seq-specific function 'voom' (34,38), selecting transcripts which had a Benjamini–Hochberg adjusted *P*-value for the statistical model applied lower than 0.05. This analysis yielded a total of 5843 differentially expressed transcripts (D3 in Figure 1).

### Prediction of miR-204 targets combining RNA-Seq analysis and bioinformatics prediction

We further selected genes that displayed the expression behavior in our experiment consistent with them being miR-204 targets. In particular, we hypothesized that direct targets would be significantly down-regulated in the OE condition and up-regulated in the KD condition, although given the context of a whole embryo experiment a target could be observed only in one of the two conditions tested. We therefore selected as potential miR-204 targets all transcripts that were expressed at lower levels in the OE model as compared to the WT (OE/WT < 0) or expressed at higher levels in the KD model compared to the WT (KD/WT > 0). This procedure yielded a dataset of 5105 putative target transcripts of miR-204 based on RNA-Seq results (D5 in Figure 1). The human orthologs for D5 transcripts were obtained using the Ensembl database, thus obtaining 3168 human genes that were analysed for functional enrichment using the DAVID web-based tools (39) specifying as background the dataset of all the human genes resulting from D2, i.e. expressed Medaka transcripts (12 225 human genes). We selected categories enriched with an adjusted *P*-value (Benjamini correction) lower than 0.05. The complete results of the enrichment analysis are presented in Supplementary Table S2. Overall, the analysis indicated a strong enrichment for genes involved in biological processes such as nervous system development (adj. *P*-value = 4.8 × 10^−28^), neurogenesis (adj. *P*-value = 3.5 × 10^−18^) and in particular in the KEGG pathway ‘axon guidance’ (adj. *P*-value = 1.42 × 10^−28^) (panel B in Figure 1 and Supplementary Figure S3a and b).

Furthermore, we retrieved a dataset of predicted miR-204 direct targets in the human genome by using three bioinformatics microRNA target prediction tools (see Materials and Methods for more details) and then we used the Ensembl BioMart data-mining tool to identify their Medaka orthologs. Using this approach we found 3720 Medaka transcripts as potential miR-204 direct targets. The overlap between transcripts predicted to be direct targets of miR-204 and differentially expressed Medaka transcripts with the expected behavior was of 785 genes corresponding to 595 human orthologs. The functional enrichment pointed, once again, to categories related to nervous system development (adj. *P*-value = 3.01 × 10^−9^), neurogenesis (adj. *P*-value = 6.45 × 10^−7^) and axon guidance (adj. *P*-value = 3.55 × 10^−9^) (Supplementary Table S3).

**Figure 2.**
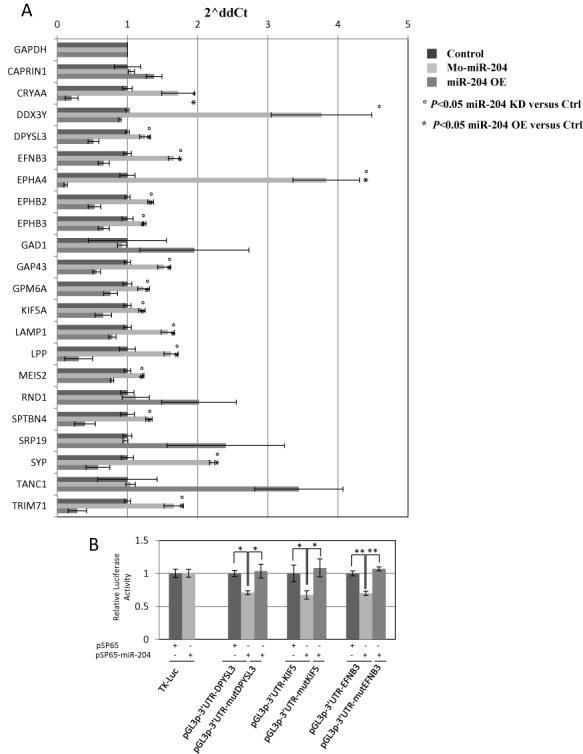
EFNB3 is a direct miR-204 target. (A) Analysis by qRT-PCR on total Medaka RNA of 21 predicted miR-204 targets from DS4. Black bars, control Medaka embryos; light gray bars KD Medaka embryos; dark gray bars, over-expression Medaka embryos. X-axis: fold change variation expressed as 2-ΔΔCt values; Y-axis: predicted target genes tested; all genes for which a decreased expression in over-expression embryos was observed as compared to KD embryos are labeled by an asterisk. The housekeeping genes used to normalize the expression of the predicted targets are *Hprt* and *Gapdh*; the errors bars are calculated on the biological duplicates. (B) Relative Luc activities as fold differences in the Luc/Renilla ratios normalized to the value of Luc reporter constructs. miR-204 addition significantly decreases Luc activity of the constructs containing 3′-UTR of *DPYSL3*, *KIF5* and *EFNB3* when compared with controls. *P < 0.05 (*t*-tests). **P < 0.0001 (*t*-tests). Three point mutations in the predicted miR-204 target sites inhibit this effect (no significant variation when compared with the thymidine kinase (TK)- Luc control).

We then selected 21 differentially expressed genes for validation. Most of the genes were chosen randomly from within the list and a few were added from those belonging to the enriched pathways (see Supplementary Table S7). We tested them in triplicate by qRT-PCR on total RNA derived, respectively, from WT, miR-204 over-expressing and miR-204 morphant Medaka embryos. We found that 76% (16/21) of the analysed genes confirmed their behavior as putative miR-204 targets with expression changes concordant with those detected by RNA-Seq (Figure 2A). To further assess the validity of qRT-PCR results, we performed dual Luciferase reporter assays focusing on four putative targets: *KIF5*, *DPYSL3*, *DDX3* and *EFNB3*. To this end, we cloned the 3′-UTR of these genes, comprising the miR-204 target site, downstream of the Luc reporter gene. MiR-204 specifically inhibited reporter expression of three out of four analysed constructs, i.e. those for the *EFNB3*, *KIF5* and *DPYSL3* genes (Figure 2B and data not shown). This effect was no longer observed when constructs with point mutations in the corresponding miR-204-binding sites were used (Figure 2B), indicating that miR-204 directly and specifically targets these transcripts.

In order to verify the conservation of the miRNA target sequences in transcripts, we reconstructed the 3′UTR starting from RNA-Seq data (Supplementary Figure S1). We integrated Ensembl annotated data with our own sequencing information to identify UTRs as they were truly transcribed in our own dataset. As reported in Supplementary Figure S1, the alignment data show an extension downstream of the last exon compared to the standard notation, demonstrating that using standard annotation is not sufficient to verify the conservation of the miRNA target sequences in the UTRs of these transcripts.

### Analysis of miR-204 targets based on rank product correlation with putative targets

**Figure 3.**
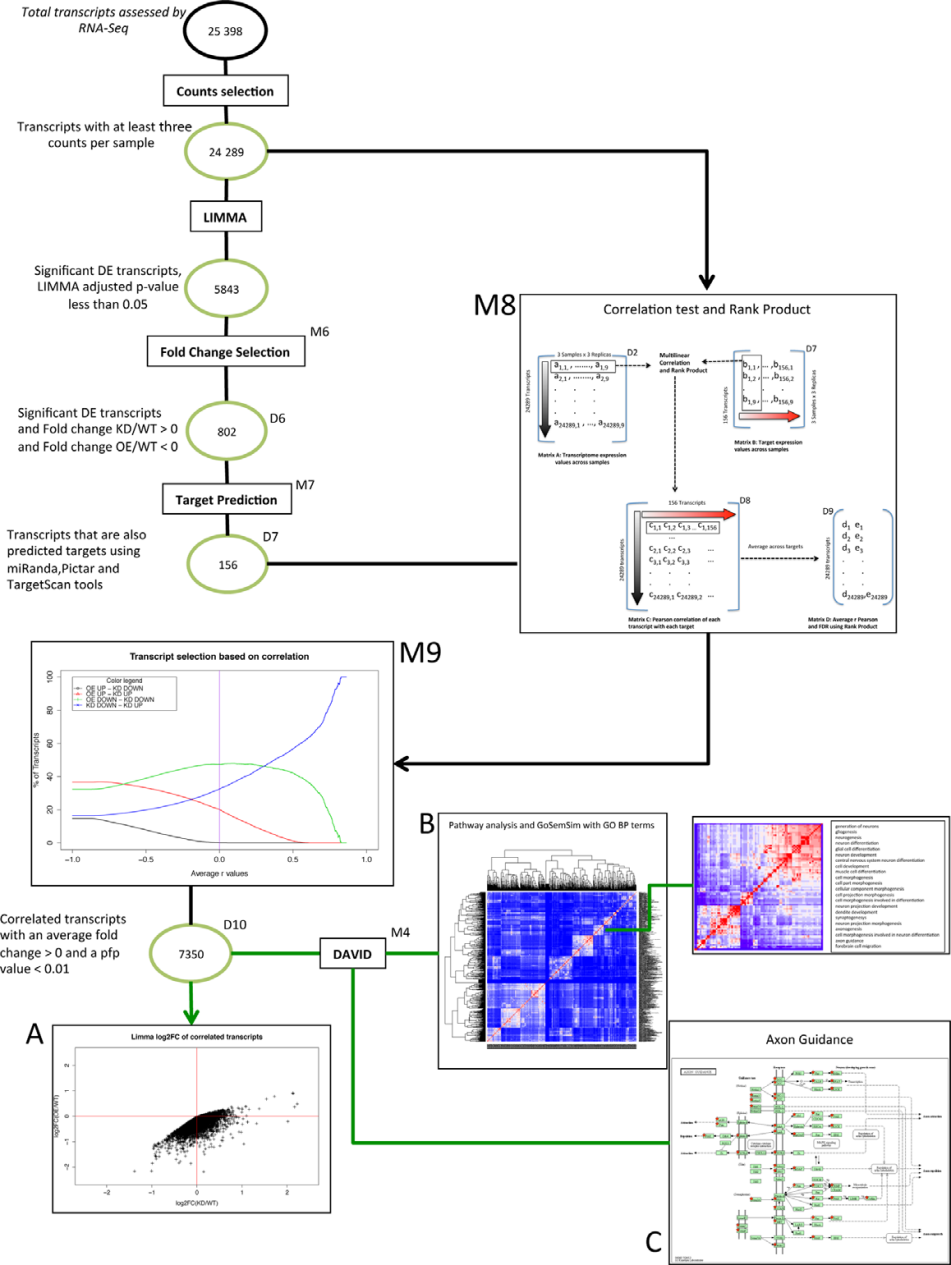
Flow chart of DGE analysis using correlation analysis and Rank Product. Circles represent datasets used in the analysis workflow, rectangles indicate methods applied to the datasets and panels present results from specific datasets. Following the transcript filtering and differential expression analysis described in Figure 1, we selected genes from D3 in a more stringent manner, by selecting transcripts behaving stringently as miR-204 targets (M6: OE/WT < 0 and KD/WT > 0) and which were predicted to be miR-204 targets by at least one bioinformatics tool (M7), yielding D7. We applied a Pearson's correlation test using normalized read count correlating D2 with D7. The result, D8 (shown in M8 panel) is a matrix of correlation values of 24 289 rows and 156 columns. Applying Rank Product statistics to D8 (shown in M8 panel), we obtain the average correlation value for each transcript in D2 based on D7, resulting in D9 (shown in M8 panel). As shown in M9 as the average Pearson's coefficients increase, the proportions of transcripts behaving as expected in OE and KD experiments increase. Transcripts with an average Pearson's correlation coefficient above 0 (purple line) and a *pfp* <0.01 were selected for further analysis resulting in D10. Panel A presents the Limma log2 fold changes for D10 transcripts, showing KD/WT values in the X-axis and OE/WT values in the Y-axis. Panel B presents Gene Ontology clusters based on DAVID functional enrichment (M4) and GOSemSim clustering. Panel C presents the Axon Guidance pathway indicating with red stars genes from D10.

We also developed an alternative approach for the identification of miRNA target genes and pathways based on gene expression correlation (Figure 3). The hypothesis on which the method is based is that the expression levels of transcripts that are direct targets of the same microRNA should correlate in the samples under investigation and that this correlation can be utilized as a tool to predict other novel targets, if appropriately analysed (20). As described above, we selected transcripts for their adjusted *P*-value and their fold change in KD or OE condition. For this analysis, we decided to seed the correlation from a stringent dataset of putative targets by applying more stringent conditions. Specifically, we took into consideration only transcripts which have the expected behavior in both conditions (KD and OE) and which are also predicted as targets by bioinformatics tools. This approach led to a set of 156 transcripts corresponding to 116 human orthologous genes (D7 in Figure 3), which was used to seed the correlation. To determine the levels of correlation within our predicted targets subset, we applied Pearson's correlation to all nine samples in the experiment through all three conditions. This approach allowed us to assess correlation at individual sample level, thus taking into account potential differences between replicas (e.g. due to different strength of effect of the KD and OE reagents or due to slight differences in cell numbers for target tissues). A hierarchical cluster analysis on these putative targets revealed that the transcripts have a high correlation amongst themselves (Supplementary Figure S4), as expected. Thus we used all 156 transcripts for further correlation analysis (D7 in Figure 3). We then tried to identify additional transcripts whose expression levels were highly correlated to the predicted target dataset. This was achieved by applying Pearson's correlation to two matrixes of expression levels across all samples: the matrix of 156 putative targets and the matrix with all expressed transcripts (see M8 in Figure 3). Using a statistical method based on the ranking of coefficients, i.e. Rank Product, we were able to identify those transcripts whose correlation with the 156 targets was the highest by applying an FDR *P*-value based on Rank Product statistics (35). Having obtained a rank product correlation value for each transcript, we were able to verify the behavior of transcripts across OE and KD experiments at different thresholds of average correlation. As the correlation threshold is increased, most transcripts behave as expected in the OE experiment, and only at the highest correlation levels do they start also behaving as expected in the KD experiment (see M9 in Figure 3 and Supplementary Figure S5). To be consistent with the LIMMA analysis described before, we thus selected transcripts which behaved as targets in OE or KD experiments, i.e. with an average Pearson's correlation coefficient for the 156 targets higher than 0 (i.e. a positive correlation with the putative targets) and a percentage of false prediction (*pfp*) value less than 0.01 (see M9 and Panel A in Figure 3).

**Figure 4.**
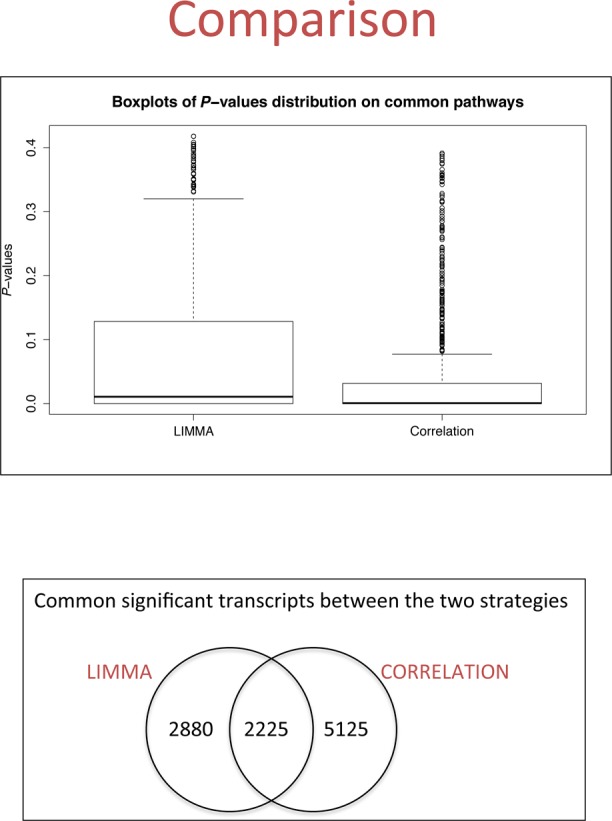
Comparison between LIMMA and CORRELATION analysis approaches on differentially expressed genes. (**A**) Boxplots of the distribution of *P*-values obtained for common pathways identified with the two strategies. For illustration purposes, we presented all common pathways and not only significant ones. (**B**) A Venn diagram on the transcripts identified by the two strategies.

We thus obtained a total of 7350 Medaka transcripts, (D10 in Figure 3) corresponding to 4133 human genes, highly correlating to *bona fide* putative targets. We performed pathway analysis on these genes using the same background described above, and we observed similar categories and pathways that were found after using LIMMA-selected transcripts supporting our previous results. For example, the following significant enrichments were observed: nervous system development (adjusted *P*-value = 3.22 × 10^−54^), neurogenesis (adjusted *P*-value = 2.75 × 10^−26^) and axon guidance (adjusted *P*-value = 2.04 × 10^−46^) (Supplementary Table S4). Overall, the correlation approach allowed us to identify more functional enrichments than the LIMMA approach. The total number of significant Gene Ontology Biological Process (GO BP) Terms and KEGG pathways identified by LIMMA approach is 721 while in the correlation approach it is 985. Moreover, on the specific enrichments that were common to both approaches (580 pathways), the correlation approach yielded stronger statistical significance (Wilcox Test *P*-value = 1.61 × 10^−27^), as shown in Figure 4.

**Figure 5.**
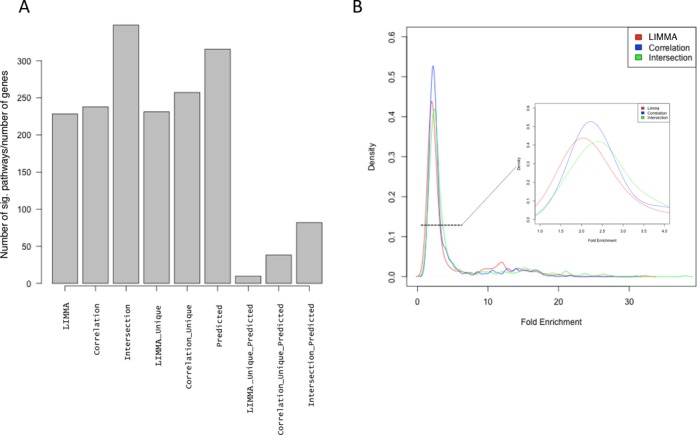
Contribution of correlation to significant pathways enrichment. (**A**) Number of significantly enriched Gene Ontology Biological Processes and KEGG pathways. The number of categories has been corrected by the number of genes in the list used to generate the enrichment. (**B**) Density plot of the distribution of Fold Enrichment of significantly enriched pathways using LIMMA (red), correlation (blue) and the intersection (green) of both approaches. (INSET in B) Zoom into largest population of pathways ranging between 1-fold and 4-fold enrichment.

In order to further explore the advantages of adding the correlation analysis to the approach, we additionally looked at the number of GO BP Terms and KEGG pathways identified by genes produced only with one or the other approach (LIMMA-unique, Correlation-unique), with those in common (intersection), with predicted targets (predicted) and the overlapping of the first three with predicted targets (Figure 5). Since the number of genes used for calculating pathway enrichment vary significantly across lists (few hundreds to few thousands) and may affect the statistics of the enrichment significantly, we compared the number of significant pathways correcting for the number of genes in the list (Figure 5A). While both approaches reached more or less the same corrected number of pathways, when used as complete sets and as unique sets, the list with genes coming from both approaches (intersection) reached the maximum number of pathways per gene. The list of predicted targets reached a higher number than both approaches separately but lower than their combination. Despite a dramatic reduction in the number of pathways enriched found when overlapping RNA-Seq-derived lists with the predicted targets, we still could see an improvement by using the correlation approach.

In addition, when looking not only at the number of pathways but also at the fold enrichment, the correlation approach and its combination with LIMMA, tended to perform better also in terms of the mean fold enrichment of pathways, as seen by the distribution of fold enrichments using LIMMA, correlation and the intersection (Figure 5B).

To better describe the potentials of using the correlation analysis, we took a look at the top pathways that were produced when using genes coming only from the correlation analysis (Supplementary Table S5). Interestingly, ‘Axon guidance’ and ‘Axonogenesis’, as well as other neuron-related pathways appeared as the most significantly enriched pathways. Indeed, when the genes in the ‘axon guidance’ KEGG pathway were inspected in terms of the approach used (Supplementary Figure S6), we could observe that while LIMMA pointed to 11 predicted genes, the Correlation pointed to 22, only 8 of them in common. Hence, by using the correlation approach in combination with LIMMA, we could expand to 14 new putative target genes within the highly enriched ‘Axon guidance’ pathway.

### *In vivo* validation of miR-204-mediated control of retinal axon guidance

The analysis described above pointed to a putative and previously unreported role of miR-204 in the regulation of axon guidance molecules. More than half of the transcripts associated with the axon guidance pathway (25/46) and with a significant score in the LIMMA or Correlation analysis, corresponded to either Ephrin ligands or EPH receptors (see Supplementary Table S6). Two members of these axon guidance molecules (40–44), *EphB2* and *Efnb3*, are *bona fide* miR-204 targets, as recently reported by Ying et al. (45) or shown here (Figure 2B). Both *EphB2* and *Efnb3* seem to control retinal axon outgrowth during vertebrate eye development (46).

**Figure 6.**
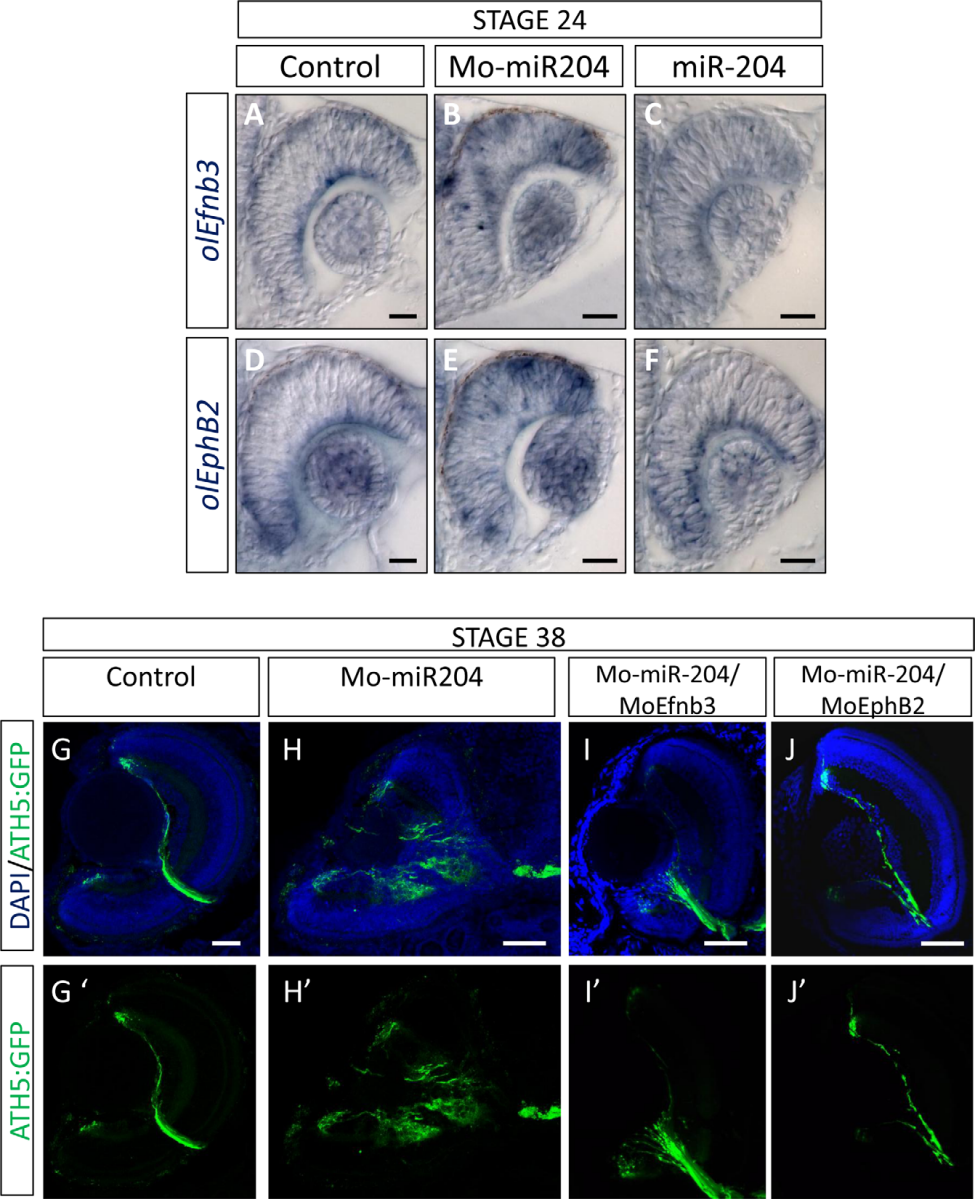
Alteration of miR-204 activity results in axon guidance defects. (**A–F**) Frontal vibratome sections of St24 control (**A and D**), miR-204 KD (**B and E**) and miR-204 over-expressing (**C and F**) embryos hybridized in whole mount RNA ISH with the *olEfnb3*- and *olEphB2*-specific probes, respectively. The expression domains of both *olEfnb3* and *olEphB2* are expanded in miR-204 KD (**B and E**), and reduced in miR-204 over-expressing embryos (**C and F**) when compared to control embryos (**A and D**). (**G–J′**) Frontal cryostat sections of St38 control (**G and G′**), miR-204 KD (**H and H′**), Mo-miR-204/Mo*Efnb3* (**I and I′**) and Mo-miR-204/Mo*EphB2* (**J and J′**) Ath5:GFP (green) transgenic embryos. In Mo-miR-204-injected embryos, RGC axons are dispersed into the retina and do not form a proper fasciculated optic nerve as seen in *Ath5:EGFP* transgenic fish (green signal) (**G–H′**). Mo-miR-204/Mo-Efnb3 and Mo-miR-204/Mo-EphB2 co-injections restore correct optic nerve and RGC axon formation (**I–I′and D–D′**).

We thus further explored the functional role of miR-204 in the regulation of *EphB2* and *Efnb3* function during the outgrowth of retinal ganglion cell (RGC) axons in Medaka fish. First, we compared the distribution of the *Efnb3* and *EphB2* transcripts in WT, miR-204 KD and miR-204 OE Medaka embryos by RNA *in situ* hybridization. We found a significant expansion of the retinal expression domains of both *Efnb3* and *EphB2* in KD compared to control injected embryos (Figure 6A, B, D and E). Conversely, we observed a reduction of their expression domains in miR-204 OE embryos when compared to controls (Figure 6A, C, D and F). Previous studies in mice showed that the proper dosage of Ephrin/EPH signal is critical for correct intra-retinal axon guidance and that EphrinB/EphB signaling is required for dorsal-ventral retinotopic targeting of RGC axons (46). We thus asked if change in miR-204 activity mimicked this phenotype. To this end, we used a Medaka transgenic line with an Enhanced Green Fluorescent Protein (EGFP) reporter under the control of the regulatory region of *Ath5* tgíAth5::eGFP, a transcription factor expressed in RGCs, since it enables to follow the complete trajectory of the growing RGC axons (29). A large proportion (65%) of miR-204 KD embryos analysed at St.38 (8 days pf) showed RGC axon pathfinding defects as determined by comparing un-injected, control and miR-204 KD olAth5:eGFP transgenic embryos. In miR-204 KD transgenic embryos, EGFP-positive RGC axons do not extend along the optic fiber layer but grow aberrantly into other retinal layers (Figure 6G, G′, H and H′). Furthermore, axons, instead of presenting a fasciculated organization as in WT embryos, were rather de-fasciculated in both the optic disk and nerve of miR-204 KD embryos (Figure 6H). Consistent with these observations, miR-204 OE *Ath5:eGFP* transgenic embryos presented an abnormal optic nerve trajectory at St. 34 (5 days and 1 h pf) (Supplementary Figure S7). In these embryos, RGC axons approached the midline but subsets of axons misprojected to the telencephalon or failed to reach the appropriate topographic target at the tectum (Supplementary Figure S7). Axon misprojections were not a consequence of defects in RGC specification because (i) *Ath5* was normally expressed at St26 (2 days and 6 h pf), i.e. when the retina begins to differentiate (9) and (ii) Pax6, a marker of both amacrine cells and RGCs was correctly expressed at later stages of retinal development in both miR-204 KD and OE embryos (Supplementary Figure S8 and data not shown).

To define if these defects were a consequence of alterations in the expression of the miR-204 targets, *Efnb3* and *EphB2*, we performed a series of rescue experiments. Indeed, the concomitant injection of Mo-Efnb3 and Mo-miR-204 (90 ± 5%, 218 out of 241 embryos; Figure 6I and I′) or that of Mo-Ephb2 and Mo-miR-204 (63 ± 5%, 183 out of 282 embryos; Figure 6J and J′) rescued the axon guidance phenotype of morphant embryos. Furthermore, the simultaneous overexpression of miR-204 and the *EphB2* mRNA, lacking the entire 3′UTR including the miR-204-binding site, resulted in embryos with normal RGC projections (71 ± 7%, 200 out of 283 embryos; Supplementary Figure S7), indicating that *EphB2* counteracted the effect of miR-204.

All in all, these data demonstrate that miR-204-mediated control of the Ephrin-EPH signaling system regulates retinal axon outgrowth, revealing a previously unidentified role for miR-204 in this developmental event.

## DISCUSSION

We demonstrated that the integration of RNA-Seq transcriptome analysis, bioinformatics approaches and *in vivo* gene manipulation in a fish model is an efficient approach to investigate the function of a specific miRNA, both by allowing an efficient recognition of their relevant targets and the identification of their role in the control of specific functional pathways. In particular, the RNA-Seq procedure and analysis approach we described in this study was instrumental to uncover a role of miR-204 in the control of retinal axon guidance, which was further confirmed by *in vivo* miR-204 KD and OE in appropriate transgenic lines. A great deal of information on miRNA biology was recently obtained by exploiting microarray-based transcriptome analyses, which were carried out using different strategies (20,21). Our study further highlights the advantages of performing such studies using techniques with much higher resolution over microarrays such as RNA-Seq, in particular in model organisms that have limited microarray platforms available. The improvement and the parallel reduction in cost of next-generation sequencing-based procedures have led to the availability of RNA-Seq-based approaches, which allow us to gain further insight into miRNA biology as well as better definition of UTR structure and usage in the developmental stage under investigation. However *in silico* analysis of this kind of data is not easy and there are no standard pipelines that can be used to reliably identify microRNA targets, so far.

In this study, we used two different approaches, a conventional differential expression approach based on the LIMMA tool, and a novel method developed by us based on correlation to validated targets. These methods were used to identify putative miRNA-204 target pathways as well as direct target genes. A direct comparison of the transcripts identified by two methods highlights that the two strategies reported qualitatively similar results in terms of pathways enrichment. On the other hand, the correlation method was able to recover gene lists with stronger statistical significance for common enriched terms, as shown in Figure 4, despite the larger number of genes identified. The correlation approach allows the identification of key genes in pathways which were not seen using the LIMMA approach and moreover, some validated targets reported in the literature but which had not initially been identified in the LIMMA analysis were found using the correlation approach. For example *Meis2*, which we previously validated as a key miR-204 target (9), was found using the correlation approach but was not identified by the LIMMA analysis. The reason is because its adjusted *P*-value in the LIMMA analysis was higher than 0.05 for all the transcripts associated with the gene, however fold change values for KD and OE were as expected, up-regulated in KD and down-regulated in OE and highly correlated to other targets.

It is widely accepted that direct miRNA targets have their levels regulated in a fine-tuning fashion with fold changes that usually do not exceed 1.1–1.3. In that scenario, many potential targets could be penalized when analysed through classical statistical tests as LIMMA whose significance strongly depends on the extent of variation. On the other hand, simply reducing stringency in the statistical analysis based on LIMMA would not necessarily yield useful results. Using the correlation analysis, we are being less stringent in looking at fold changes because it scores that fine-tuning behavior that takes into account not only perturbations of miRNA levels in the experiment but also minimal baseline variance within biological replicates due to effect strength of the KD or OE models, fine developmental staging differences or cell numbers for specific target tissues. However, false discovery correlations are filtered out by using a consistent group of inter-correlated targets as input and by applying a false discovery correction based on non-parametrical Rank Product statistics. By using the combination of the two approaches, we take advantage of correlation to uncover a higher number of putative targets, specially those with very small changes that may not pass the classical significance filters of state-of-the-art RNA-seq differential expression tools as LIMMA while focusing our attention on those with the expected biological behavior.

Such an approach is still affected by some caveats and could be improved in further settings. Our study has been conducted in whole Medaka embryos. It is of course clear that the expression of miR-204 could be restricted to some tissues and areas and hence the KD experiment would work as expected only as far as the miRNA is highly expressed in those tissues with respect to the whole embryo. Moreover, it can be argued that transcriptome analysis on well-defined tissues is advisable to identify targets more clearly; however, utilizing whole embryo has allowed us to identify targets expressed across both ocular and non-ocular tissues of great importance for visual function and axon guidance. Finally, our study is only able to address microRNA targets, which are regulated at the transcript level. Recent studies have demonstrated the importance of translation inhibition as a mode of microRNA targeting (47,48), which cannot be addressed through standard RNA-Seq approaches, but require ribosome profiling, which was beyond the scope of this study.

Despite the availability of high-throughput gene expression data, we still lack a standard method that could use this data to aid in the identification of true miRNA targets amongst the in-silico predicted based on seed sequences. Yet, some attempts have been successfully applied assuming the fact that targets of the same miRNA tend to be co-expressed and have their expression levels correlated, either to find targets of a given miRNA (20) or to find groups of genes whose mRNAs compete for the same pool of miRNAs (49). In this study, we have applied a modification of the basis of the COMETA approach (20), which used a large number of expression datasets in different tissues, to a reduced RNA-seq dataset, showing that the procedure works also when used with much smaller datasets.

Recently, emerging evidences highlight a direct and prominent role for the miRNAs in the control of neurite outgrowth, spinal morphology and function during nervous system development (50). In most of these examples, miRNAs are reported to regulate responsiveness to signaling from adjacent tissues by acting on the local regulation of gene expression and protein synthesis in axons (51). Here, we provide evidence that, in addition to these mechanisms, proper navigation of vertebrate RGC axons in the visual system development could depend on miR-204-mediated regulation of both Ephrin ligand and EPH receptor gene networks, highlighting a novel aspect of miRNA-regulated axon growth control. MiR-204 together with other genes participating to the regulation of retinal axon guidance is strongly expressed in the differentiating RGC from St.26, when the axons from those cells are forming and outgrowing outside the retina (9). Confirming and extending our previous studies on the key role of miR-204 in eye development, we show here that down-regulation of miR-204 causes abnormal growth of RGC axons in the retina. Conversely, aberrant projection to the contralateral optic nerve and ectopic rostral extension of visual fibres through the telencephalon are the most prominent abnormalities we observed in miR-204 OE Medaka embryos. Therefore, our data show that miR-204 is required for proper axon growth of RGC *in vivo* through, at least in part, the targeting of some Eph receptors, such as *EphB2* (45) and of some of their Ephrin ligands, including the newly identified target *Efnb3*.

In conclusion, by integrating transcriptome, existing and novel bioinformatics approaches, *in vitro* and *in vivo* validations, we were able to explore and further define a key player in axon guidance, mir-204, and reveal novel target genes and pathways of this microRNA.

## SUPPLEMENTARY DATA

Supplementary Data are available at NAR online.

SUPPLEMENTARY DATA
