# Supplementary material for: The combination of transcriptomics and informatics identifies pathways targeted by miR-204 during neurogenesis and axon guidance
Source: Nucleic Acids Res. 2014 Jun 4;42(12):7793–806. doi: 10.1093/nar/gku498 (PMC4081098; doi:10.1093/nar/gku498)
Supplement: SUPPLEMENTARY DATA [file supp_42_12_7793__index.html]

The combination of transcriptomics and informatics identifies pathways targeted by miR-204 during neurogenesis and axon guidance — SUPPLEMENTARY DATA 

# The combination of transcriptomics and informatics identifies pathways targeted by miR-204 during neurogenesis and axon guidance

## SUPPLEMENTARY DATA

**Files in this Data Supplement:**

- SUPPLEMENTARY DATA
- SUPPLEMENTARY DATA
- SUPPLEMENTARY DATA
