# Supplementary material for: The combination of transcriptomics and informatics identifies pathways targeted by miR-204 during neurogenesis and axon guidance
Source: Nucleic Acids Res. 2014 Jun 4;42(12):7793–806. doi: 10.1093/nar/gku498 (PMC4081098; doi:10.1093/nar/gku498)
Supplement: SUPPLEMENTARY DATA [file supp_gku498_nar-02440-y-2013-File008.pdf]

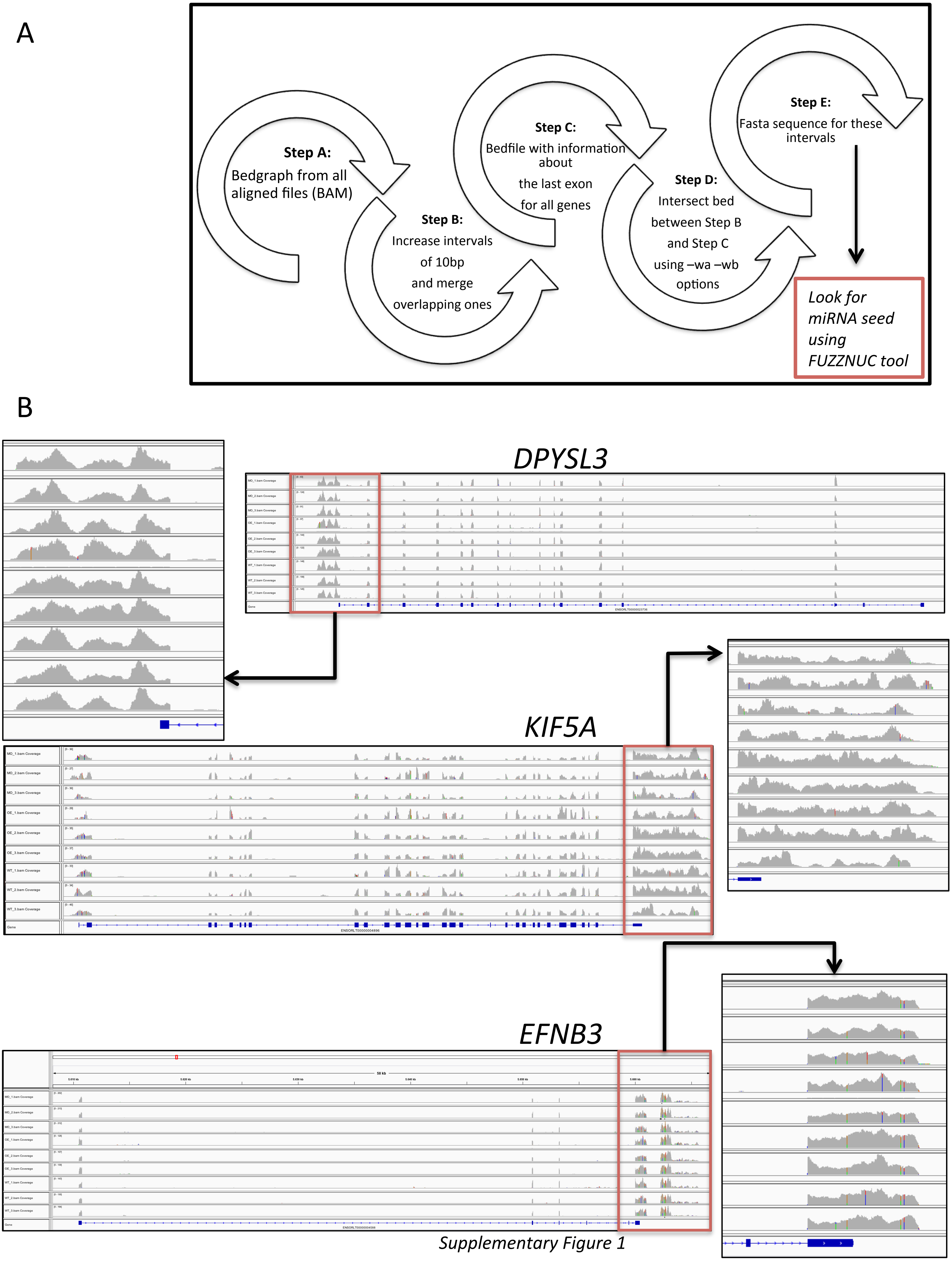

Supplementary Figure 1

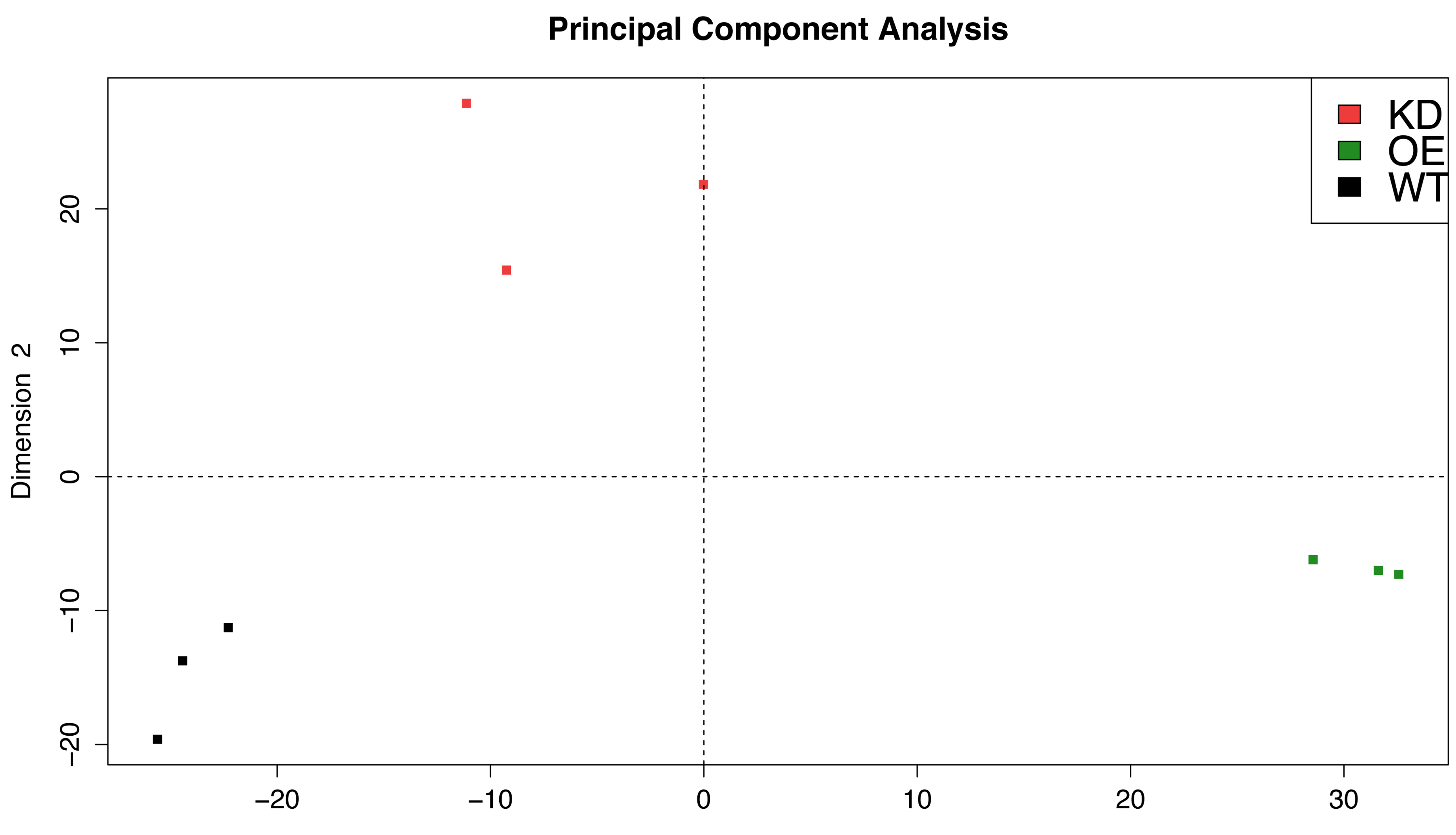

Supplementary Figure 2

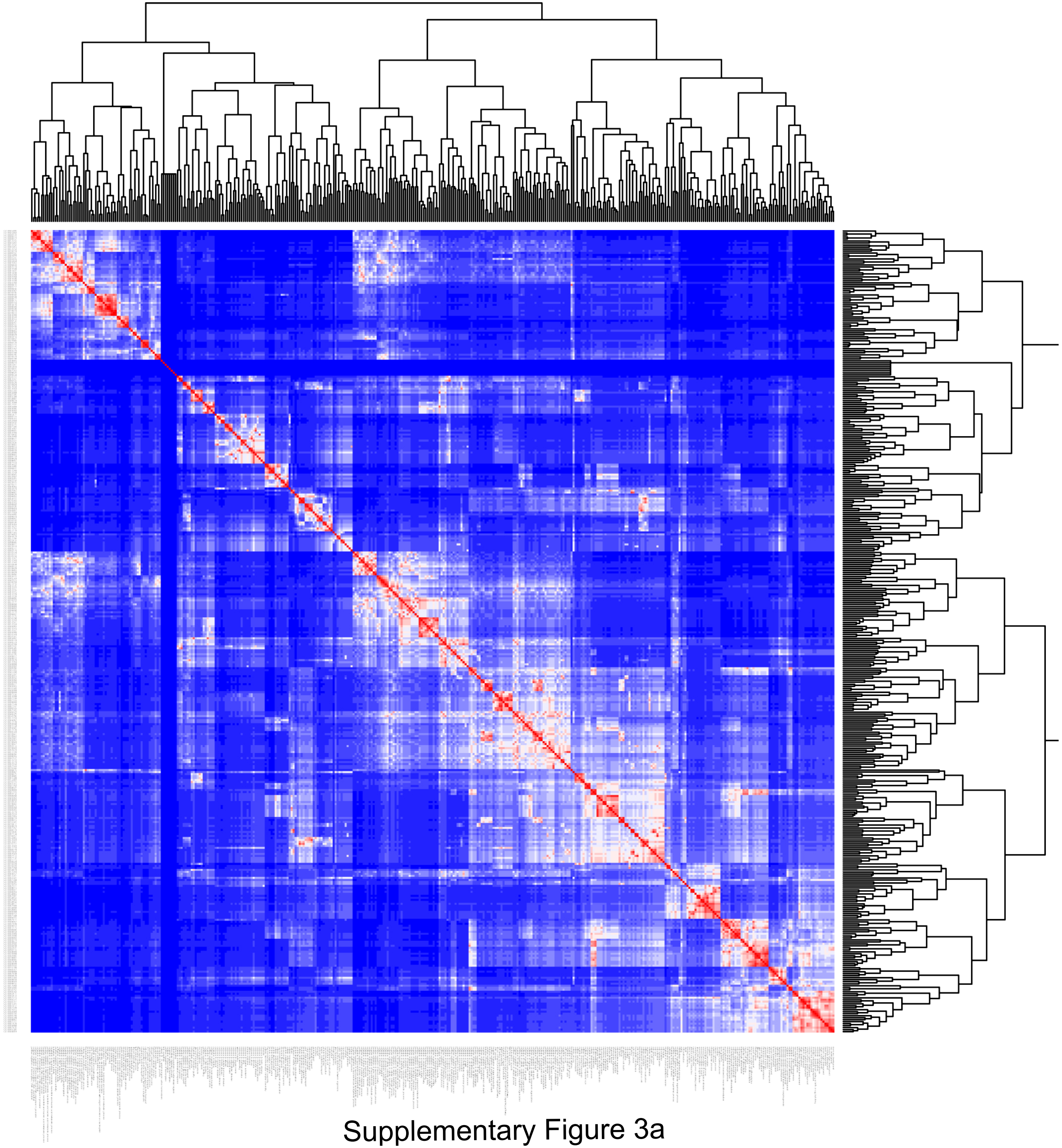

Supplementary Figure 3a

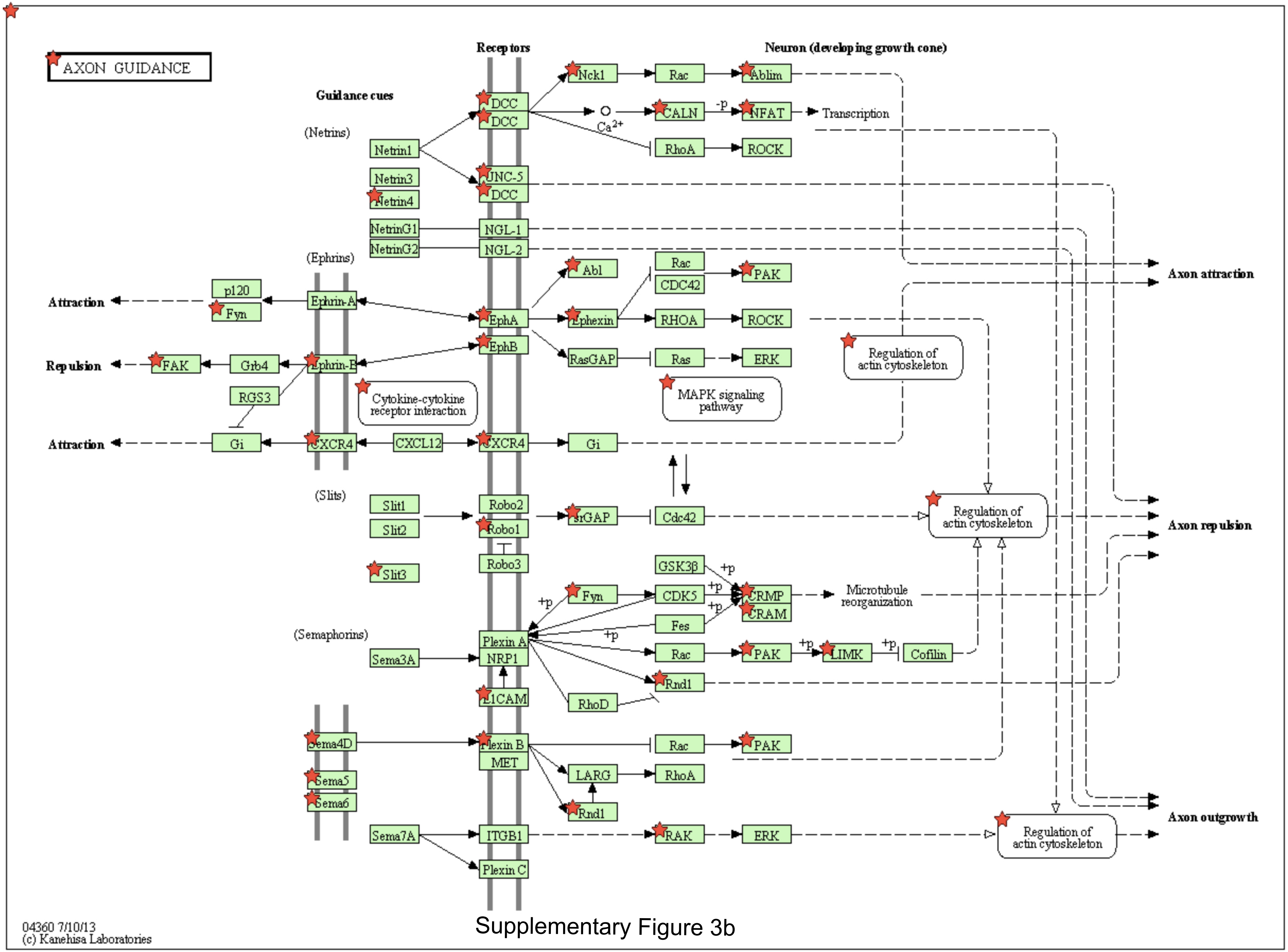

Supplementary Figure 3b

Supplementary Figure 4

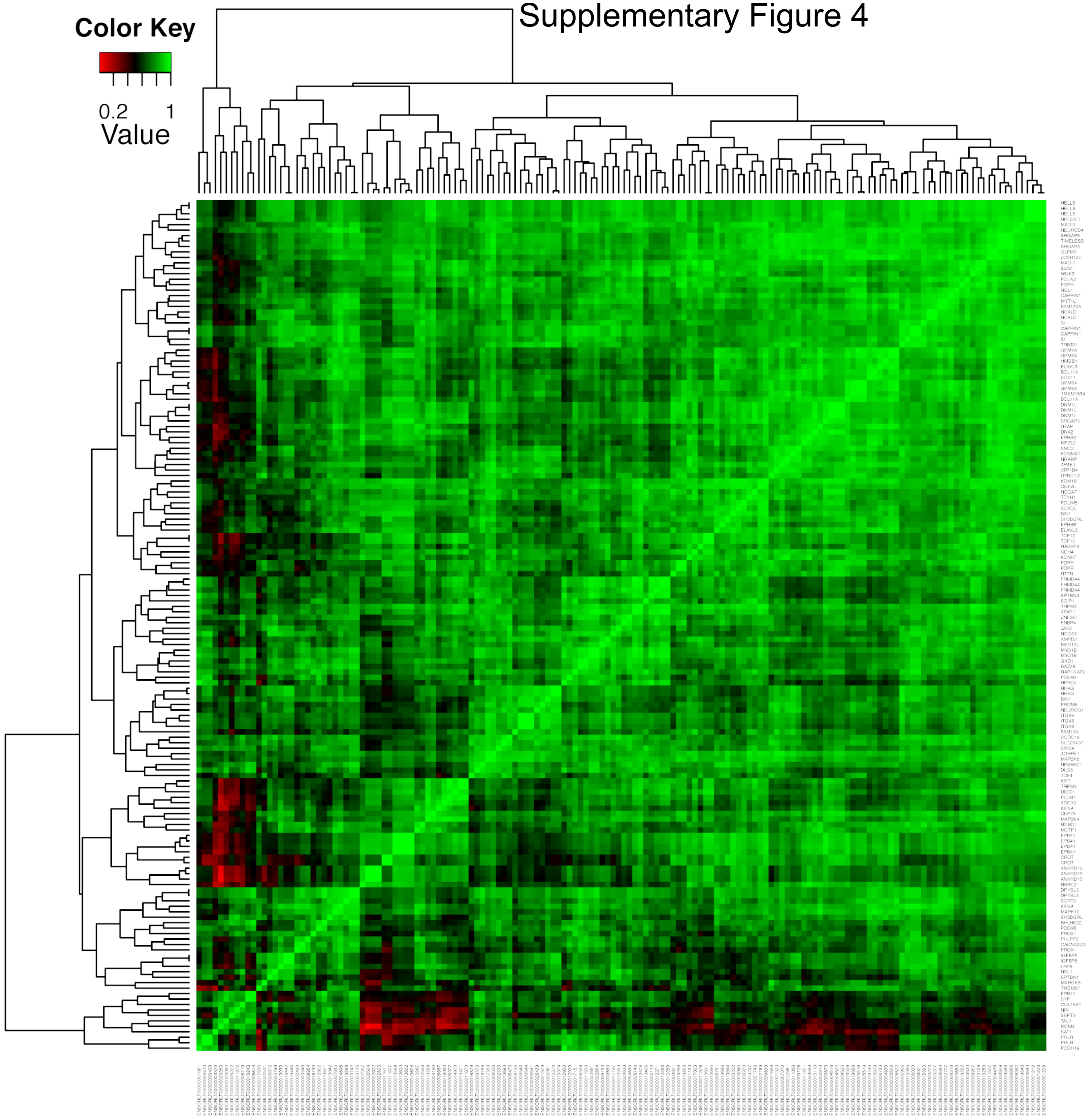

Transcript selection based on correlation

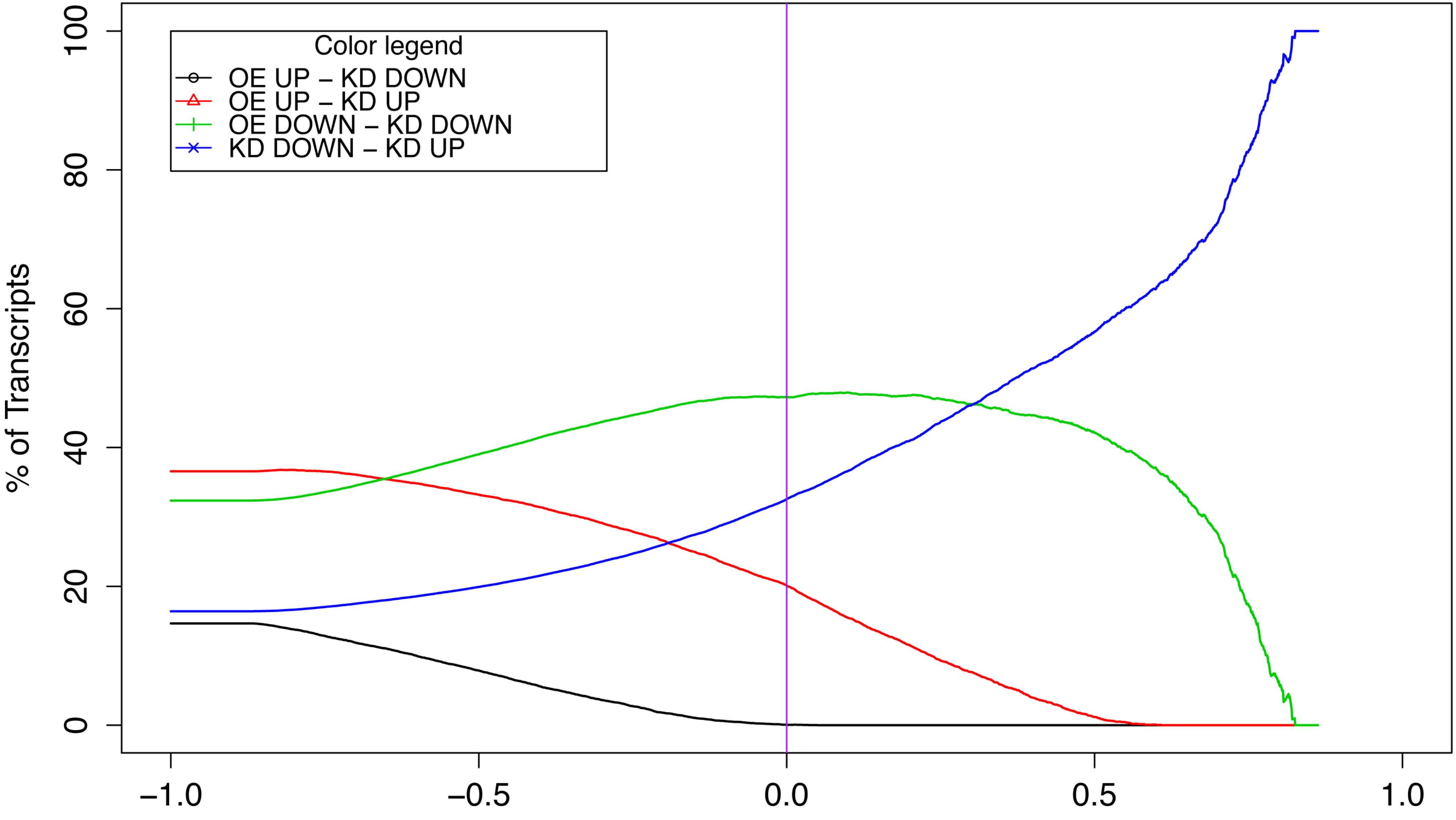

Average r values
Supplementary Figure 5

Venn Diagram of Axon Guidance Genes based on Approach

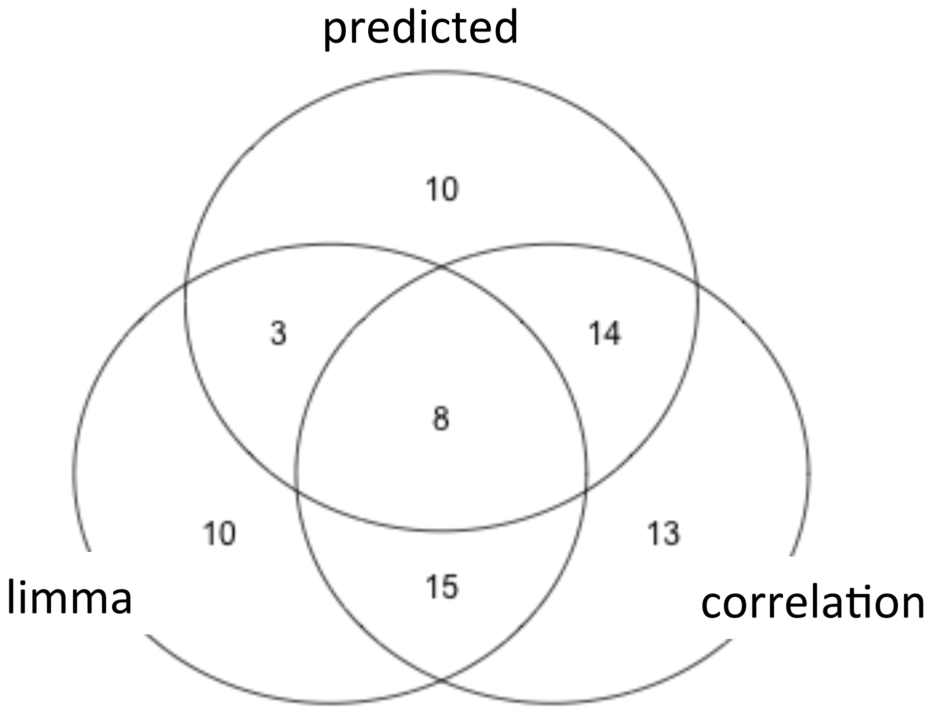

Supplementary Figure 6

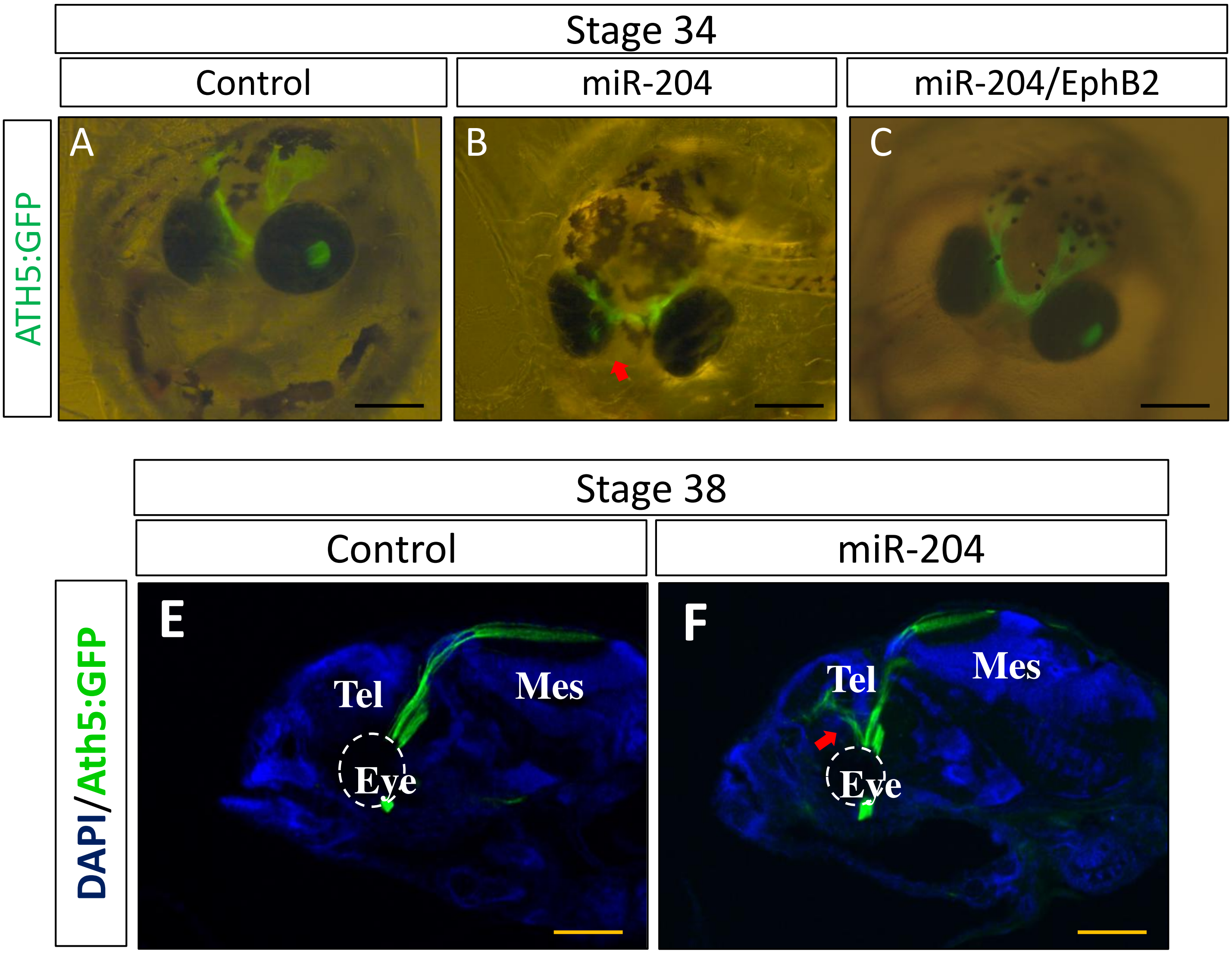

Supplementary Figure 7

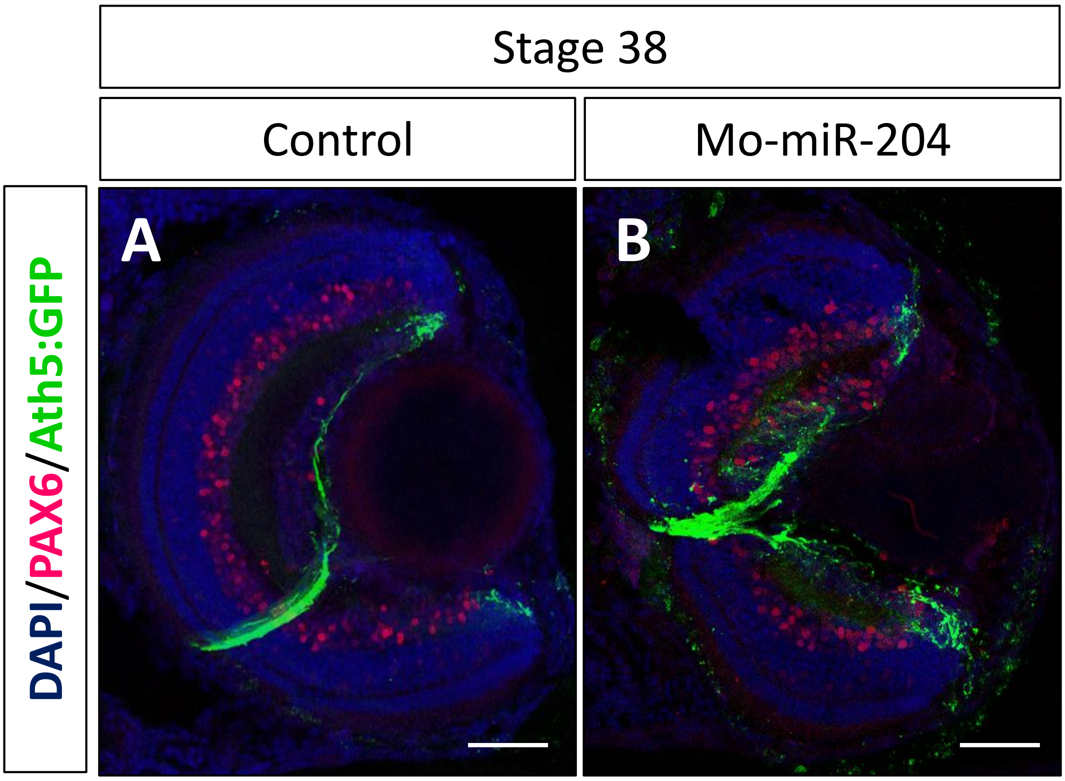

Supplementary Figure 8
