# Supplementary material for: The combination of transcriptomics and informatics identifies pathways targeted by miR-204 during neurogenesis and axon guidance
Source: Nucleic Acids Res. 2014 Jun 4;42(12):7793–806. doi: 10.1093/nar/gku498 (PMC4081098; doi:10.1093/nar/gku498)
Supplement: SUPPLEMENTARY DATA [file supp_gku498_nar-02440-y-2013-File010.docx]

**Supplementary Figure 1: Bioinformatics 3’ UTR reconstruction**

1. Flowchart of bioinformatics process used to reconstruct the 3’ UTR of Medaka transcripts using the RNA-Seq data. With UCSC BEDTools we generated a unique bedgraph file from all aligned samples, merging together the overlapping intervals. We also generated a separated bed file containing the coordinates of the last exon of all annotated transcripts. We then intersect these two files to retrieve information about the coordinates of the transcribed and sequenced region downstream the last exon. We convert this last file with extended updated coordinates in a FASTA file containing the nucleotide sequences. Using a tool for motif search we were then able to identify the miRNA seed sequences in these extended transcripts.
2. 3 examples of genes in which the standard annotation lacks of information about the 3’ UTR, which is recovered through UTR reconstruction from RNA-Seq data. For each gene the annotated transcript is reported (blue) and the RNA-Seq coverage from all BAM files (grey). The plot revels an extension downstream of the last exon compared to the standard annotation. The plots are generated using the IGV tool.

**Supplementary Figure 2: Principal Component Analysis**

Two-dimensional Principal Component Analysis on RNA-Seq samples expression values. It is clear that the transcriptome profiles of WT animals is differentiate from both miR-204 Over-Expression and Knock-Down samples.

**Supplementary Figure 3: Pathway analysis on LIMMA selected genes**

1. Estimation of Gene Ontology semantic similarities on those genes identified using LIMMA approach. The analysis revealed a strong enrichment for genes involved in biological processes such as nervous system development. In the figure they are represented by red clusters.
2. KEGG Axon Guidance pathway. The red stars highlights the genes differentially expressed identified by LIMMA approach and present in the pathway.

**Supplementary Figure 4: Hierarchical cluster analysis on putative targets**

The hierarchical cluster is performed on those genes identified by LIMMA approach and intersected with those genes predicted by bioinformatics tools as direct target of miR-204. It reveals that the transcripts have a high correlation amongst themselves. The selected transcripts were than used for Correlation approach as described in the text.

**Supplementary Figure 5: Transcription selection based on correlation**

Plot showing the dependence of average R values of Pearson correlation with known targets on the percentage of transcripts with different RNA-Seq fold-change behaviours between WT, KD and OE conditions (different colours in legend). We selected for further analyses transcripts with an average R Pearson correlation coefficient above 0 (purple line) and a percentage of false prediction value lower than 0.01.

**Supplementary Figure 6: LIMMA and Correlation approach comparison**

Venn Diagram of genes in ‘Axon Guidance’ KEGG pathway found using genes produced by miRNA target prediction alone, limma, and correlation approaches.

**Supplementary Figure 7: Alteration of miR-204 activity results in axon guidance defects by misregulation of the “Ephrin/EPH receptor” pathway.**

(A-C) Lateral views of the optic nerve of control (A), miR-204- (B) and miR-204/*EphB2*-injected (C) olAth5::GFP transgenic embryos where visual fibers are marked by GFP. In miR-204-injected embryos, RGC axons (marked by red arrowhead in F) project to the telencephalon where they find their incorrect topographical location. MiR-204/EphB2 co-injections restore correct RGC axonal projections and the optic nerve properly reaches the optic tectum (C). (E-F) Lateral cryostat sections of St38 control and miR-204-overexpressing Ath5:GFP transgenic embryos. In miR-204-injected embryos, RGC axons (marked by red arrowhead in D) are projected to the telencephalon where they find their incorrect topographical location. Sections are counterstained with DAPI (blue). Lateral views of the optic nerve of control (E) and miR-204 (F) injected olAth5:eGFP transgenic embryos where visual fibers are highlighted by GFP. In the miR-204 overexpressing embryos, optic nerve (marked by red arrowhead in F) fails to reach the optic tectum and RGC axons are projected to the anterior part of the brain (F).

**Supplementary Figure 8: Alteration of miR-204 activity does not alter RGC and amacrine cell specification.** (A-B) Frontal cryostat sections of St38 control and miR-204 KD Ath5:GFP (green) transgenic embryos immunostained with antibody against Pax6 (red). In Mo-miR-204-injected embryos, RGC axons are dispersed into the retina and do not form a proper fasciculated optic nerve as shown by Ath5:eGFP (green) staining; however this defect is not a consequence of defects in RGC and amacrine cell specification as shown by Pax6 immuno-staining (red).
